# Antagonism of the prostaglandin D_2 _receptor CRTH2 attenuates asthma pathology in mouse eosinophilic airway inflammation

**DOI:** 10.1186/1465-9921-8-16

**Published:** 2007-02-28

**Authors:** Lena Uller, Jesper Mosolff Mathiesen, Lisa Alenmyr, Magnus Korsgren, Trond Ulven, Thomas Högberg, Gunnar Andersson, Carl GA Persson, Evi Kostenis

**Affiliations:** 1 Dept. Experimental Medical Science, Lund University, Sweden; 2 7TM Pharma A/S, Fremtidsvej 3, 2970 Hørsholm, Denmark; 3 Dept. Clinical Pharmacology, Lund University Hospital, Lund, Sweden

## Abstract

**Background:**

Mast cell-derived prostaglandin D_2 _(PGD_2_), may contribute to eosinophilic inflammation and mucus production in allergic asthma. Chemoattractant receptor homologous molecule expressed on TH_2 _cells (CRTH2), a high affinity receptor for prostaglandin D_2_, mediates trafficking of TH_2_-cells, mast cells, and eosinophils to inflammatory sites, and has recently attracted interest as target for treatment of allergic airway diseases. The present study involving mice explores the specificity of CRTH2 antagonism of TM30089, which is structurally closely related to the dual TP/CRTH2 antagonist ramatroban, and compares the ability of ramatroban and TM30089 to inhibit asthma-like pathology.

**Methods:**

Affinity for and antagonistic potency of TM30089 on many mouse receptors including thromboxane A_2 _receptor mTP, CRTH2 receptor, and selected anaphylatoxin and chemokines receptors were determined in recombinant expression systems *in vitro*. *In vivo *effects of TM30089 and ramatroban on tissue eosinophilia and mucus cell histopathology were examined in a mouse asthma model.

**Results:**

TM30089, displayed high selectivity for and antagonistic potency on mouse CRTH2 but lacked affinity to TP and many other receptors including the related anaphylatoxin C3a and C5a receptors, selected chemokine receptors and the cyclooxygenase isoforms 1 and 2 which are all recognized players in allergic diseases. Furthermore, TM30089 and ramatroban, the latter used as a reference herein, similarly inhibited asthma pathology *in vivo *by reducing peribronchial eosinophilia and mucus cell hyperplasia.

**Conclusion:**

This is the first report to demonstrate anti-allergic efficacy *in vivo *of a highly selective small molecule CRTH2 antagonist. Our data suggest that CRTH2 antagonism alone is effective in mouse allergic airway inflammation even to the extent that this mechanism can explain the efficacy of ramatroban.

## Background

The small lipid mediator prostaglandin D_2 _(PGD_2_) is the major cyclooxygenase metabolite of arachidonic acid and is released by activated mast cells in response to allergen exposure [1]. PGD_2 _has long been considered a potentially important mediator in several diseases such as asthma, allergic rhinitis, atopic dermatitis, and allergic conjunctivitis [2-5]. PGD_2 _elicits biological responses by interaction with three specific seven-transmembrane receptors, referred to as DP/DP1, DP2/CRTH2, and TP (DP, D prostanoid receptor; CRTH2, chemoattractant receptor homologous molecule expressed on T helper type 2 cells; TP, thromboxane A_2 _receptor) [6-8]. Via interaction with one (or a combination) of its three specific receptors PGD_2 _may contribute to bronchoconstriction, eosinophilia and mucus production in allergic asthma. However, assessment of actual roles of PGD_2 _in allergic diseases has been hampered by its very short biological half-life and the lack of specific receptor antagonists suitable to uncover how signaling of individual PGD_2 _receptors contribute to disease processes *in vivo*. It is also possible that the contribution of PGD2 to allergic airway inflammation is easily missed if the load of allergen in challenge studies is too large [9].

CRTH2 is expressed on eosinophils, TH_2 _cells and basophils, which are all considered to contribute to the pathogenesis of allergic diseases [3,10-15]. Several lines of evidence suggest that activation of CRTH2 in response to PGD_2 _mediates recruitment of inflammatory cells *in vitro *and *in vivo*. *In vitro*, activation of CRTH2 induces chemotaxis of TH_2 _cells, eosinophils, and basophils [7,16]. *In vivo*, CRTH2 mediates mobilization of eosinophils from guinea-pig bone marrow [17], promotes eosinophilia and exacerbates pathology in mouse models of allergic asthma and atopic dermatitis [18], and induces eosinophil infiltration into the airways upon intratracheal administration of PGD_2 _or a selective CRTH2 agonist [19-21]. Based on evidence supporting a pro-inflammatory role of CRTH2, this receptor has attracted great interest as a drug target for therapeutic intervention in allergic diseases. Confusingly, however, allergic mice that lack a functional CRTH2 receptor and hence are incapable to signal through CRTH2 have been reported to exhibit both increased [22] and reduced [23,24] allergic inflammation in models of asthma [22-24] and atopic dermatitis [23]. These diverging reports involving gene-deficient animals further underscore the need to use specific CRTH2 antagonists to explore the *in vivo *function of CRTH2.

It was recently reported that ramatroban, which was initially developed as a TP antagonist and is now used for treatment of allergic rhinitis in Japan, also displays potent CRTH2 antagonistic activity [25]. Consistent with this finding, ramatroban has been shown to abrogate blood eosinophilia induced by a CRTH2-specific agonist in rats [19] and to inhibit PGD_2_-stimulated human eosinophil migration *in vitro *[25]. While these data are congruent with the notion that ramatroban acts through inhibition of CRTH2 receptors, it is not clear whether its clinical efficacy in allergic rhinitis is due to inhibition of TP, CRTH2 or both receptors. We have recently reported a ramatroban analog (given internal code number TM30089) with high antagonistic potency on and selectivity for human CRTH2 and devoid of affinity to the TP receptor [26,27].

Here we report that TM30089 is a highly potent antagonist on mouse CRTH2 and lacks affinity to mouse TP in agreement with its pharmacological properties on the human receptor orthologs. In addition, this study demonstrates that TM30089 does not display appreciable affinity to a range of other receptors and enzymes that have potentially important roles in allergic asthma. Furthermore, using a mouse model of allergic asthma involving two allergen challenges, we have discovered that TM30089 similar to ramatroban inhibits allergen challenge-induced airway tissue eosinophilia and mucus cell hyperplasia. These *in vivo *data support the promise of CRTH2 as a target for treatment of allergic airways diseases.

## Methods

### *In vitro *analysis

#### Cloning and expression of the mouse CRTH2 (mCRTH2) and mouse thromboxane A_2 _(mTP) receptor in HEK293 cells

The coding sequence of mCRTH2 (genbank accession no. AF054507) was amplified by PCR from mouse hippocampus cDNA and inserted into the pcDNA3.1(+) expression vector (invitrogen). A HEK293 cell line, stably expressing mCRTH2 (hereafter referred to as mCRTH2-HEK cells) was generated under G418 (Gibco #11811) selection and used as described below in whole cell binding and functional inositol phosphate accumulation assays. The coding sequence of mTP (genbank accession no. D10849) was amplified by PCR from mouse spleen cDNA and inserted into pcDNA3.1(+). HEK293 cells were transiently transfected with mTP as described [27] and assayed after 48 hr as outlined below.

#### mCRTH2 and mTP whole cell binding

Binding assays were performed essentially as described previously [27]. In brief, mCRTH2-HEK cells or HEK cells transiently transfected with mTP were seeded into white 96 well plates (Costar #3917) at a density of 30.000 cells/well. About 18–24 hr later, whole cell competition binding experiments were performed using 0.8 nM [^3^H]PGD_2 _(Amersham #TRK734, 166 Ci/mmol) or 0.7 nM [^3^H]SQ29548 (PerkinElmer #NET-936, 48.2 Ci/mmol), for mCRTH2 and mTP respectively. Total and nonspecific binding were determined in the absence and presence of 10 μM PGD_2 _for mCRTH2 and 10 μM U46619 for mTXA_2 _receptors.

#### Inositol phosphate accumulation assays

mCRTH2-HEK or HEK cells transiently transfected with mTP were seeded at a density of 30.000 cells/well in poly-D-lysine coated 96-well tissue culture plates and labeled by overnight incubation in medium containing 5 μCi *myo*- [2-^3^H]-inositol (TRK911, Amersham Biosciences). After washing, cells were stimulated with the respective ligands in the presence of 5 mM LiCl (HBSS, GIBCO cat. 14025–050, 45 min, 37°C) and the inositol phosphate fraction was quantified using a scintillation proximity assay (SPA) as described previously [28]. To facilitate inositol phosphate generation by the Gi-selective CRTH2 receptor, mCRTH2-HEK cells were transiently transfected with a promiscuous Gα protein [29].

#### TM30089 profiling panel

TM30089 was tested at a final concentration of 10 μM in either binding or functional assays on several 7TM receptors, and in enzymatic assays on selected enzymes including those involved in arachidonic acid metabolism. All assays were performed using recombinant human receptors and enzymes, and included the appropriate controls for assay validation. Enzymatic and binding assays, except for muscarinic binding, were performed by CEREP in vitro pharmacology profiling services. Full description of methods and references is available on the CEREP Web site [30]. Muscarinic binding assays were performed on mouse brain homogenate (non-selective) or porcine heart membranes (M2-selective) with 0.2 nM 3H-NMS as a tracer and atropine as control. Functional assays were performed using recombinant human receptors and enzymes, and included the appropriate controls for C3a, C5a, ChemR23 receptors by determining the inhibitory potency of TM30089 in InsP assays essentially as described above. For stimulation of C3a, C5a, and ChemR23 receptors in InsP assays the following agonists were used: human recombinant C3a (C3a receptor), human recombinant C5a (C5a receptor), and the nonapeptide Chemerin (ChemR23).

#### Data analysis

was performed using the Prism 3.0 software (GraphPad Software, San Diego) as described previously [27] with the following exceptions: Binding data from competition binding assays were normalized to percent of specific binding of [^3^H]PGD_2 _or [^3^H]SQ29548 and the K_i _was then determined using the Cheng-Prusoff equation *K*_*i *_= IC_50_/(1+*L*/*K*_*d*_), where *L *is the concentration of radioactive ligand and K_d _is its dissociation constant. For Schild analysis, global fitting using the Gaddum/Schild competitive interaction model was applied after the functional concentration-response curves for the agonists PGD_2 _or U46619 in the absence or presence of various antagonist concentrations were normalized to percent of the maximal response obtained by agonist alone.

### *In vivo *model of allergic asthma

#### Animals

Female BALB/c mice about 6 weeks of age (MoB A/S; Ry, Denmark) were kept in well-controlled animal housing facilities and fed *ad libitum*. The study was approved by the Regional Ethics Committee in Malmoe-Lund, Sweden.

#### Allergen sensitization and challenge protocol

All groups of mice (n = 10 in each group) were immunized by intraperitoneal (i.p) injections of ovalbumin (OVA, 10 μg per injection; grade V, Sigma, St Louis, Mo) absorbed to alum adjuvant (Al(OH)_3_; Sigma) at day 0 as described previously [31,32]. Fourteen days after immunization mice were exposed to aerosolized OVA (1 % wt/vol) 30 minutes daily for 2 days. Control animals received saline challenge. Animals were sacrificed 24 hr after the last allergen challenge by pentobarbital i.p followed by bronchoalveolar lavage (BAL) and lung tissue sampling. In preliminary studies it was ascertained that daily allergen challenges for only a few days sufficed to produce a significant airway tissue eosinophilia as well as goblet cell hyperplasia.

#### Treatment with CRTH2 antagonists in vivo

On days 14 and 15 mice were treated twice daily with TM30089 and ramatroban (BAY3405), 5 mg/kg orally. Control animals received vehicle (PBS 10% Tween80). Drugs were administered 30 minutes before and 4 hr after each challenge. The dose of ramatroban was selected in accord with previous reports [20,23]. Furthermore, ramatroban and TM30089 differ chemically merely by the move of one carbon atom from the carboxyl side chain in ramatroban to the sulfonamide nitrogen in TM30089 – thus they have the same molecular weight and other important structural features in common – so it is highly reasonable to assume they will have very similar PK properties. Hence, based on the additional similarity regarding CRTH2 antagonism *in vitro *the same dose of the TM30089 was used in the in vivo part of this study to allow for a head-to-head comparison.

#### Staining and quantification of lung tissue eosinophils and mucus-containing cells

Eosinophils were detected by histochemical visualization of cyanide-resistant eosinophil peroxidase (EPO) activity [33]. In brief, 5 μm cryo sections were incubated for 8 min at room temperature in PBS buffer (pH 7.4) supplemented with 3.3-diaminobenzidine tetrahydrochloride (60 mg/100 ml; Sigma), 30% H_2_O_2 _(0.3 ml/100 ml), and NaCN (120 mg/100 ml). Slides were then rinsed in tap water and mounted in Kaisers medium (Merck, Darmstadt, Germany). Eosinophils were identified by their dark brown reaction product and eosinophils were counted around bronchi using a depth of 120 μm from the epithelial basement membrane and expressed as cells/0.1 mm^2 ^tissue area as previously described [32]. 5 μm cryo sections were stained with periodic acid-Schiff reagent (PAS) and the total number of PAS-positive cells counted and expressed as cells/mm basement membrane as previously described [32]. Although the focus in this study was on tissue pathology BAL was also carried out (see below).

#### Bronchoalveolar lavage (BAL) and quantification of luminal cells

Although eosinophils, according to the preliminary studies, clearly had migrated to the airway tissue the brevity of present allergen challenge period until termination meant that the tissue eosinophils had only started to be lost into the airway lumen. However, it was still of interest to examine BALF eosinophils to see if drug-induced effects on tissue eosinophilia in part could be due to increased loss of these cells through migration across the epithelial lining [34]. BAL was performed via a ligated tracheal cannula and 1 ml of PBS was allowed to passively enter the lungs at a pressure of 10 cm H_2_O, a procedure that is gentle to the lungs [32]. The obtained BAL-fluid (BALF) from each animal was immediately centrifuged and the supernatant frozen for ELISA analysis. The total number of cells was quantified using a cell sorter (NucleoCounter^®^, Chemometec A/S, Allerod, Denmark) and 5 × 10^5 ^cells cytocentrifuged to microscope slides. Differential cell counts were performed on May-Grünwald Giemsa stained slides and percentage of eosinophils, lymphocytes, neutrophils, and macrophages determined by counting 200–300 cells in a blinded manner. To obtain the absolute number of each leukocyte subtype in each BALF, the percentage of cells was multiplied by the total number of cells recovered by the BAL.

#### Data analysis

Histology analyses were performed and quantified in a blinded manner. To compare histological data, the non-parametric Kruskal-Wallis test and followed by Connover's test was applied using using Analyze It™ (Analyse-it software Ltd., Leeds, UK). Data are expressed as mean ± SEM. A value of p < 0.05 was considered statistically significant.

## Results

### In vitro pharmacological profile of TM30089 and ramatroban

**Figure 1:**
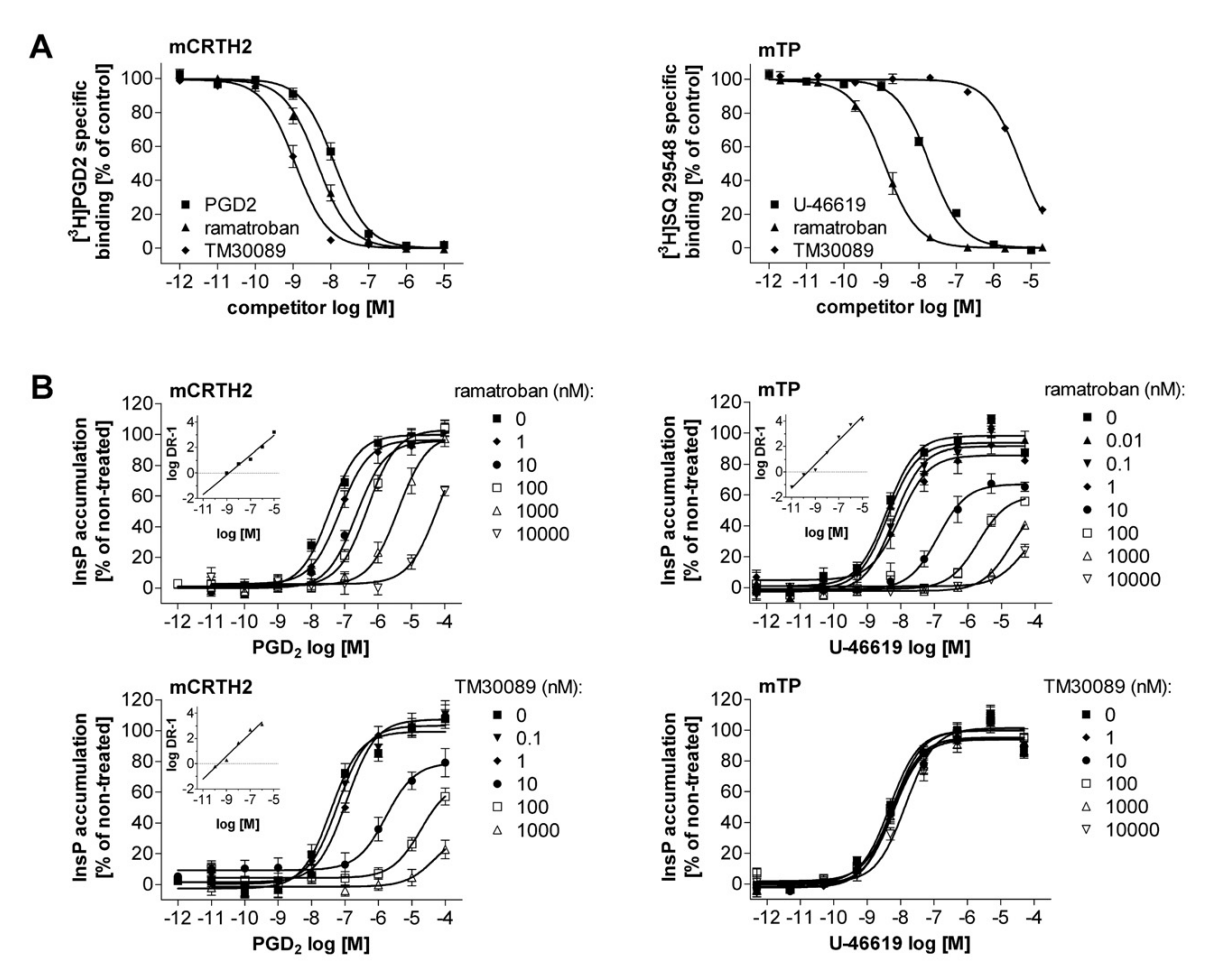
**In vitro characterization of compounds on mouse CRTH2 (mCRTH2) and mouse TP (mTP) receptors**. **A**, Competition binding analysis. **B**, inhibition of mCRTH2 and mTP receptor function. PGD_2_- or U-46619-concentration response curves in the absence and presence of the indicated compounds in mCRTH2- or mTP-expressing cells. Inserts: Schild plots. Schild analysis show potent antagonism of mCRTH2 by TM30089 (pA_2 _= 9.15 ± 0.11, Schild slope = 1.45 ± 0.08) whereas it does not interfere with signaling of mTP. Ramatroban is a potent antagonist on mCRTH2 (pA_2 _= 8.08 ± 0.14, schild slope = 0.94 ± 0.05) and mTP (pA_2 _= 9.36 ± 0.10, Schild slope = 1.35 ± 0.06). Experiments show mean ± SE from 5–7 independent experiments.

We have recently reported that the ramatroban analog TM30089 displays high affinity to human CRTH2 (hCRTH2) but completely lacks affinity to the human thromboxane A_2 _receptor hTP (TP, T prostanoid receptor), unlike ramatroban which antagonizes both receptors equally well [27]. To investigate whether TM30089 represents a suitable tool compound to explore the role of CRTH2 in a mouse model of allergic inflammation *in vivo*, we cloned the mouse orthologs of CRTH2 and TP and evaluated TM30089 and ramatroban (as a reference) in their ability to displace [^3^H]PGD_2 _and ^3 ^[H]SQ29548 from mCRTH2 and mTP, respectively, in competition binding experiments using human embryonic kidney cells expressing the individual receptors. Both compounds displayed high affinity to mCRTH2 with log pK_i_= 8.96 ± 0.05 (1.1 nM), for TM30089 and pK_i_= 8.38 ± 0.05 (4.2 nM) for ramatroban (FIG 1A). The affinity of ramatroban in our binding assay is in good agreement with the reported K_i _value of 28 nM obtained by others in the same expression system [35]. In contrast to ramatroban which also displayed high affinity to mTP (pK_i_= 8.92 ± 0.05 (1.2 nM)), TM30089 bound to mTP with only negligible affinity (pK_i_= 5.30 ± 0.03 (50.000 nM)). Thus, both compounds retain their selectivity profile for CRTH2 versus TP on the mouse receptor orthologs: TM30089 is CRTH2-selective, while ramatroban is a dual CRTH2/TP ligand (FIG 1A).

To confirm antagonistic efficacy of the compounds their ability to inhibit cellular signaling of the receptors was evaluated in a functional second messenger assay (FIG 1B). In agreement with the binding data, TM30089 acts as a potent antagonist on mCRTH2 (pA_2 _= 9.15 ± 0.11, Schild slope = 1.45 ± 0.08) but does not interfere with signaling of mTP, as compared with ramatroban which potently inhibits cellular signaling of both mCRTH2 (pA_2 _= 8.08 ± 0.14, Schild slope = 0.94 ± 0.05) and mTP (pA_2 _= 9.36 ± 0.10, Schild slope = 1.35 ± 0.06) receptors (FIG 1B). To ascertain that *in vivo *efficacy of TM30089 does not arise from inhibition of related 7TM receptors such as the anaphylatoxin receptors C3a and C5a, the chemokine receptors CCR3 and ChemR23, or the second high affinity PGD_2 _receptor DP, it was tested for its affinity or antagonistic potency on these receptors (Table 1). Notably, TM30089 is very selective, exhibiting > 1000-fold preference for CRTH2 over DP [26], and lacks affinity to any of the other tested receptors and also to the two cyclooxygenase isoforms 1 and 2 which are recognized as important players in allergic airway inflammation [36,37]. Together, the affinity and selectivity profile of TM30089 suggests that it may be suited to uncover the *in vivo *contribution of CRTH2 in allergic airway inflammation.

**Table 1:** Profiling of TM30089 at relevant 7TM receptors (binding or functional assays) and enzymes (enzymatic assays).

Receptor	Assay type^a^	% of control^b^
AT_1_	B	90.1 ± 2.8
AT_2_	B	98.1 ± 1.4
BLT_1_	B	89.3 ± 3.5
C3a	F	88.7 ± 5.9
C5a	B	108.7 ± 6.3
ChemR23	F	96.6 ± 1.6
CXCR2	B	104.6 ± 6.1
CysLT_1_	B	116.9 ± 10.5
Glucocorticoid	B	98.9 ± 11.3
Muscarinic (non-selective)	B	106.7 ± 8.5
Muscarinic (M2-selective)	B	97.5 ± 1.9
Phospholipase A_2_	E	101.5 ± 1.0
COX_1_	E	109.6 ± 3.6
COX_2_	E	123.8 ± 13.4
iNOS	E	102.3 ± 3.6

^a ^Assay type: B – radioligand binding, F – functional InsP, and E – enzymatic assays.

^b ^Assays were performed in the absence (100% control) or presence of 10 μM TM30089 (% of control). Results are given as % of control ± SE (estimated) from 2–3 independent experiments.

### In vivo effects of CRTH2 antagonists on asthma-like airway histopathology

**Figure 2:**
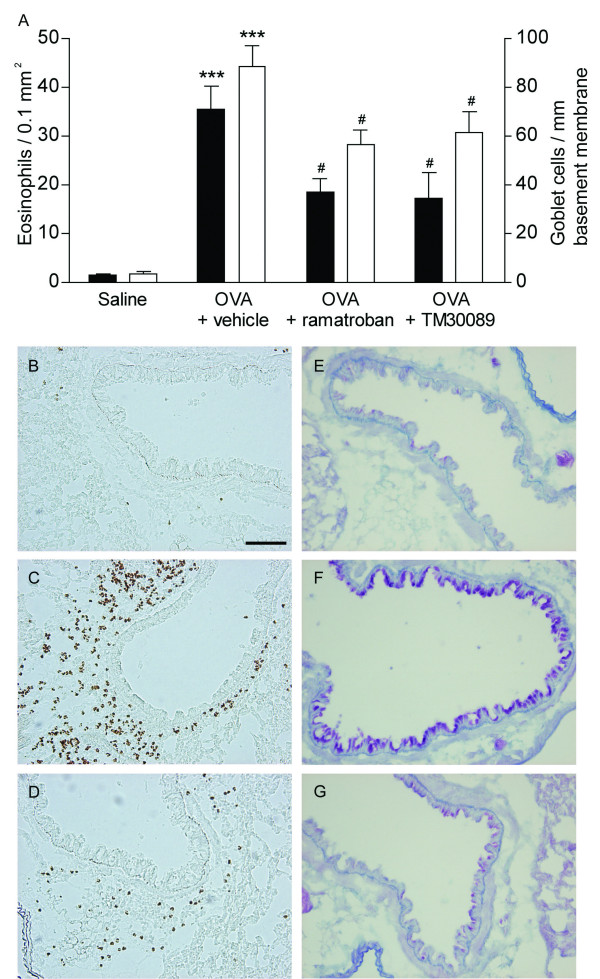
**CRTH2 antagonism in vivo attenuates airway tissue eosinophilia and mucus cell hyperplasia**. Above (A): OVA challenge produced marked eosinophilia and mucus cell hyperplasia (***, p < 0.001 compared to saline control). Treatment with ramatroban or the specific CRTH2 antagonist TM30089 significantly reduced airway tissue eosinophilia (black bars) and mucus production (white bars); ^#^, p < 0.05 compared to vehicle treatment. Below (B-G): Light micrographs showing effects in particularly well-responding animals. Lung tissue eosinophilia in normal saline treated lung (B), OVA/vehicle treated lung (C), and OVA/TM30089 treated lung (D). Airway mucus cells are shown in normal saline treated lung (E), OVA/vehicle treated lung (F), and OVA/TM30089 treated lung (G). Scale bar = 100 μm.

Mouse models of allergic asthma exhibit some of the cardinal histopathological signs of human asthmatic lungs, most notably eosinophilia and mucus cell hyperplasia [32,38]. These are also the key features investigated in this study. Mice were immunized with the allergen (OVA) and challenged two times by inhalation of aerosolized OVA. Lung histology was assessed 24 hr after the last challenge. OVA-challenged mice developed a marked peribronchial lung tissue eosinophilia (35.5 eosinophils ± 4.7/0.1 mm^2^) p < 0.0001 compared to saline challenged animals (1.3 ± 0.3 cells) (FIG 2a). Eosinophilia was accompanied by a significant increase of mucus cells in the airway mucosa (88.1 ± 8.8; 3.2 ± 1 cells/mm basement membrane), (FIG 2a). Treatment with TM30089 and ramatroban significantly diminished the allergen challenge-induced peribronchial lung tissue eosinophilia and mucus cell hyperplasia (FIG 2). Inhibition by both compounds of pathological changes was quantitative and did not alter the overall pattern of occurrence of eosinophils and mucus cells in the airway-lung tissues (FIG 2).

Elimination of the airway tissue eosinophils largely occurs through egression of these cells into the airway lumen [32,39,40] where they can be retrieved by BAL. As indicated by the BALF eosinophilia (1.0 ± 0.2; 0.02 ± 0.02 % eosinophils in allergen and saline-challenged animals, respectively), eosinophils had already started to enter into the airway lumen about 24 hr after the second allergen challenge. Both drug treatments tended to reduce BALF eosinophilia (TM30089 0.5 ± 0.01 %; ramatroban 0.4 ± 0.01 % BALF eosinophils) indicating that the attenuation of tissue eosinophilia induced by these drugs did not reflect increased elimination of tissue eosinophils into the airway lumen.

## Discussion

Emerging evidence suggests that PGD_2_-activation of its high affinity receptor CRTH2 may be particularly critical in the pathogenesis of eosinophilic airway inflammation, since activation by PGD_2 _of CRTH2 potently stimulates chemotaxis of eosinophils *in vitro *and *in vivo *[7,19-21,25,41-43]. Herein, we demonstrate that the small molecule TM30089 is a highly potent and selective inhibitor of mouse CRTH2 function *in vitro*, and by using this antagonist demonstrate for the first time that inhibition of CRTH2 signaling *in vivo *suppresses the development of certain key features characteristic for allergic asthma.

*In vitro*, TM30089, which is structurally closely related to ramatroban, was found to bind mouse CRTH2 with nanomolar affinity and potently inhibit its signaling in cells overexpressing the receptor. In addition, TM30089 completely lacks affinity to the mouse TP receptor, unlike ramatroban which represents a dual TP/CRTH2 antagonist on both human and mouse receptor orthologs [25,26] (this study). Furthermore TM30089 in concentrations up to 10 μM did not show significant binding to or inhibition of selected chemokine receptors, anaphylatoxin receptors, the other high affinity PGD_2 _receptor DP as well as the cyclooxygenases 1 and 2. Owing to its high selectivity over all other tested receptors and enzymes, and the fact that its chemical structure is closely related to ramatroban which has proven efficacious in various animal and human studies of allergic rhinitis and asthma [44-50], TM30089 emerges a suitable research tool to explore the contribution of CRTH2-signaling in allergic airway inflammation *in vivo*.

We found that administration of TM30089 displayed anti-inflammatory efficacy in a mouse model of allergic asthma that mimics some of the major histopathological features characteristic of human asthma such as eosinophilia and mucus cell hyperplasia [38]. In the present study we used allergen challenges during only two consecutive days which proved to be sufficient for causing goblet cell hyperplasia as well as a significant accumulation of eosinophils in airway-pulmonary tissues. More challenges for more days would have increased the eosinophilia further. However, the present model was preferred because it had been reported that efficacy of interference with PGD_2 _is dependent on the allergen load [9]. Thus, the marked protection against allergen-induced asthma observed in DP-/- mice was completely lost when more than three allergen challenges were used [9]. It is of note that although the present number of provocations was low the load of allergen would still be far greater than the level of allergens human asthmatics are exposed to.

The present observation of increased BALF eosinophils agrees with previous data indicating that already during the build-up of tissue eosinophilia by allergen exposure these cells begin to be lost into the airway lumen [32]. Yet, studies resorting exclusively to the determination of BALF eosinophilia run the risk of drawing incorrect conclusions about changes in eosinophil numbers in the most important locale, the airway-pulmonary tissues. For example, we and others have previously observed in allergic mice that drug interventions may inhibit lumen eosinophilia whilst the tissue eosinophilia remains unchanged or is, indeed, increased as reviewed in [34]. It was, therefore, important to note in this study that both drug treatments tended to reduce BALF eosinophils demonstrating that increased elimination of these cells into the airway lumen could not explain the present inhibitory effects of these drugs on airway tissue eosinophilia. Using TM30089, we thus unravel that CRTH2-signaling appears integral to the recruitment of eosinophils to the airways *in vivo*. So far, it has only been demonstrated that externally administered PGD_2 _is able to induce local eosinophilia in different models of inflammation [18-21,51], and that CRTH2 is the cellular mediator for this effect. However, whether CRTH2 signaling is relevant in a disease paradigm has not been established to date. The present finding that a small molecule inhibitor of CRTH2 is effectively attenuating eosinophil trafficking to the airway tissues hence suggests that CRTH2 is an important effector in this OVA-induced asthma model and regulates allergic inflammation *in vivo*. Since recruitment of eosinophils to inflammatory sites is considered a critical parameter in asthma and other allergic diseases [11-13], our findings also highlight the potential importance of CRTH2 as a novel therapeutic target.

Interestingly, the selective CRTH2 antagonist TM30089 equals the dual TP/CRTH2 antagonist ramatroban regarding inhibition of eosinophil recruitment *in vivo*. Ramatroban has previously not only been shown to attenuate airway inflammation in guinea pig and mouse asthma models [45], but also been effective in inhibiting eosinophil infiltration into the nasal mucosa in patients suffering from allergic rhinitis [48]. It remained elusive, however, whether inhibition of CRTH2 or TP or both receptors accounted for its anti-inflammatory efficacy in these studies. Potentially, inhibition by ramatroban of eosinophil trafficking may be explained by two different mechanisms: (i) blockade of TXA_2_-mediated expression of adhesion molecules on endothelial cells and/or (ii) direct inhibition of CRTH2-dependent eosinophil migration [7,52]. The present finding that both, a dual TP/CRTH2 antagonist as well as a selective CRTH2 antagonist are comparable in their ability to prevent peribronchial eosinophil infiltration in OVA-sensitized mice therefore is indicative of the notion that exclusive inhibition of CRTH2 may also be sufficient to prevent eosinophil infiltration into the airway tissues in allergic humans. Thus, it is tempting to speculate that efficacy of ramatroban is likely related to inhibition of CRTH2 rather than inhibition of TP both in rodent asthma models and in humans.

Other characteristic features of allergic asthma are mucus hypersecretion and airway remodeling [3,53]. Mice challenged by inhalation of aerosolized ovalbumin showed marked goblet cell hyperplasia, which was significantly attenuated in animals treated with the dual TP/CRTH2 antagonist ramatroban or the selective CRTH2 antagonist TM30089. Interestingly, exclusive inhibition of CRTH2 by TM30089 was equally effective as compared with dual TP/CRTH2 antagonsim in ameliorating goblet cell hyperplasia. This finding is intriguing since corticosteroids, which now are mainstay asthma treatment, have variable effects on suppression of goblet cell hyperplasia [54], and offers the exciting perspective that selective inhibition of CRTH2 may be beneficial to achieve clinical improvement of allergic asthma. Further studies are warranted to investigate the molecular basis for the beneficial effects of CRTH2 antagonists in experimental allergic asthma in more detail.

Recent reports dealing with CRTH2-deficient mice have generated a highly inconsistent picture of receptor function. On one hand, OVA-sensitized mice lacking CRTH2 displayed enhanced occurrence of eosinophils in BALF [22]. Unfortunately, data on airway tissue eosinophils were not presented [22]. On the other hand and in apparent contrast, CRTH2-deficient mice from another laboratory were protected from bronchial hyperresponsiveness, and mucus production [24]. Furthermore, from a third laboratory IgE-mediated skin eosinophilic inflammation was significantly reduced in CRTH2-deficent mice compared to wild type animals [23]. These latter authors [23] also reported that they could not confirm the previously reported increase in lung eosinophilia [22] and they stated that only further studies including CRTH2 antagonists will contribute to final conclusions in this field. Although the specific contribution of CRTH2 in allergic inflammation thus is inconsistent in studies using mice with targeted gene disruption, the findings of the present study are congruent with the notion that CRTH2 represents an eosinophilotactic receptor and provide strong support for the concept that CRTH2-signaling *in vivo *is an important molecular step in the pathogenesis of allergic asthma.

It is intriguing to note that CRTH2 is not only activated by PGD_2 _and several of its metabolites the generation of which is dependent on the enzyme PGD synthase, but also by products of the arachidonic acid cascade that are generated independent of PGD_2 _production. Among the latter are the TXA_2 _metabolite 11-dehydro-TXB_2_, and PGF_2α_, that have recently been demonstrated to activate eosinophils and basophils [43, 55]. The possibility that CRTH2 can be activated in cellular contexts where PGD synthase is not present, i.e. in the absence of PGD_2 _production, further corroborates its importance as a regulator of allergic inflammation and underscores the potential usefulness of CRTH2 antagonists as anti-asthmatic agents.

## Conclusion

Our study is the first to demonstrate efficacy of a small molecule selective CRTH2 antagonist in an experimental model of eosinophilic airway inflammation, and indicates that CRTH2-signaling is integral for orchestrating some of the pathological features characteristic for allergic asthma such as recruitment of eosinophils and mucus cell hyperplasia. Although we cannot rule out at present that inhibition of both TP and CRTH2 may be superior to obtain clinical efficacy in allergic diseases, our data certainly suggest that blockade of CRTH2 alone is sufficient to yield anti-inflammatory efficacy in experimental asthma, even to the extent that this mechanism may explain the efficacy of ramatroban. Although the OVA-induced asthma model does not reproduce all the features of the human disease, we propose that selective CRTH2 antagonists represent a novel and promising therapeutic approach to treat allergic asthma and related inflammatory diseases.

## Competing interests

The author(s) declare that they have no competing interests.

## Authors' contributions

LU, JMM, LA, MK, TU, TH, GA, CGAP, EK participated in the design and performance of the study and LU, JMM, TU, CGAP and EK drafted and wrote the manuscript. All authors read and approved the final manuscript.
